# Physical activity among adolescents and barriers to delivering physical education in Cornwall and Lancashire, UK: A qualitative study of heads of PE and heads of schools

**DOI:** 10.1186/1471-2458-8-273

**Published:** 2008-08-01

**Authors:** Spencer Earl Boyle, Georgina L Jones, Stephen J Walters

**Affiliations:** 1 University of Sheffield, Health Services Research, ScHARR, Research Centre, Regent Court, 30 Regent St, Sheffield, S1 4DA, UK; 2 University of Sheffield, Institute of General Practice & Primary Care, ScHARR, Community Sciences Centre, Northern General Hospital, Herries Road, Sheffield, S5 7AU, UK

## Abstract

**Background:**

Recent initiatives have been introduced by the UK government into secondary schools to increase pupils' access to physical activity (PA). Despite this, not enough is known about pupils' levels of physical activity or whether the delivery of these initiatives in schools facilitates or creates a barrier for pupils' PA. The aim of this study was to gain an understanding of adolescents PA levels from the perspective of those responsible for delivering physical education (PE) in schools; heads of PE (HOPE) and heads of school (HS).

**Methods:**

Seventeen semi-structured qualitative interviews were carried out with a snowball sample of HOPE and HS in schools in the Northwest and Southwest of England. Thematic data analysis using NVIVO was used to identify emergent themes.

**Results:**

17 core themes were generated, 12 of which confirmed the findings from similar research. However, five themes relating to 'ethos of performance/elitism', 'lower fitness leads to lower ability', 'undervaluing activities within PE dept' or school as a whole', 'role of the school' and 'PE department doing all it can' offer valuable new insight into the factors which may encourage or prevent PA inside or outside the curriculum.

**Conclusion:**

Despite many positive perceptions of the delivery of PE in schools, it is evident that barriers still exist within that delivery which discourages physical activity. More research is needed to particularly address the complex issues of elitism and the ethos of PA in schools.

## Background

Recent data indicate that almost one in four young people in the UK (23.7% of 11–15 year old males and 26.2% females) are now classed as obese [1]. There is much speculation about the causes of obesity in young adolescents. However, it has been reported that one of the leading contributory factors of childhood obesity is a lack of physical activity (PA) [1]. Although a common standard of the optimum level of young people's physical activity has yet to be universally agreed upon [2], the UK government (as part of its physical education school sports club links strategy), set a target in 1999 that 85% of school children should take part in two hours per week of high quality sport and physical education (PE) and a variety of new initiatives were introduced in schools to help children achieve this target by 2008 [3].

Nevertheless, despite these new initiatives there is still controversy amongst physical educators and academics over whether young people are obtaining adequate levels of PE and are sufficiently physically active. For example, according to a Sport England (2003) survey of 158 PE teachers, 49% of children between the ages of 2–11 years received two hours of PE in school time per week, but 26% received less than an hour and a half [4]. However, according to Department of Health figures in England, the percentage of children receiving two hours of PE per week has risen to 69% [1].

PE teachers' task in promoting the health and activity of young people has recently been given more recognition by governmental authorities [5]. Despite this, a literature search revealed a paucity of recent research examining teachers' experiences of the delivery of PA in schools and the influence this may have on adolescents overall activity levels. However, one study in this area reported that teachers were concerned that using vigorous activity in lessons to create opportunities for pupils to increase their heart rate could compromise other PE objectives such as developing knowledge and skills [6].

Consequently, the aim of this study was to use qualitative methods to gain an understanding of adolescents PA levels from the people responsible for delivering PE in schools: namely heads of physical education (HOPE) and heads of school (HS). A qualitative methodology was chosen because whereas one of the main aims of quantitative methods is to explain causal relationships, qualitative research searches for reasons, motives or explanations [7]. Thus, the aim of qualitative research is to describe and interpret meanings and experiences of people as accurately as possible. In order to try to understand and explore HOPE and HS perceptions on factors which determine physical activity in children, qualitative research was therefore considered the best methodology to adopt for this study.

## Methods

### Sample

Ethical approval to interview the teachers was gained through the University of Sheffield's School of Health and Related Research ethics committee. To recruit HOPE and HS, a letter detailing the study was administered to all public secondary schools in the Northwest (NW) (East Lancashire) and the Southwest (SW) (Cornwall). HOPE and HS were asked to either telephone or email if they were interested in participating. This initial postal survey yielded a poor response rate. From 68 schools (37 in the NW and 31 in the SW), only four HOPE replied. Consequently, a snowball sampling methodology was used to generate the sample of interviewees [8].

The four HOPE who initially replied were contacted and these teachers^# ^suggested a further list of potential HOPE and HS to approach. This resulted in a further 40 schools being approached to participate (via the snowball recruitment method). Each teacher received details of the study and gave written consent to take part and have their interview recorded. All the semi-structured interviews were conducted at the teacher's own school at a time convenient to the individual member of staff.

(^# ^Throughout the report the term teachers refers to both HOPE and HS)

### Data collection: Interview guide

The aim of the interviews was to explore and understand the teachers' perceptions of what encouraged and discouraged children's participation in PA (both inside and outside the school). The intention was to gain a better understanding of the teachers' perspectives of provision and participation by pupils in physical activity and whether differences between regions (i.e. NW and SW) existed. The interviews were introduced with *'I am trying to find out about how your school or departments policies on provision of PE and physical activity encourage children's participation, so from your time in teaching what are your views about activity levels of children?' *A semi-structured interview guide was then used which included several prompting questions on the area of the national curriculum for PE (e.g. lesson content and timetabling) and the schools provision of activity opportunities both within and outside school hours. All the interviews were recorded and transcribed verbatim (with permission).

### Data analysis

Data analysis was carried out using NVIVO (a software programme specifically designed to analyse qualitative data). A thematic analysis of the data was used to interpret the transcribed interviews. The themes or concepts that emerged from the data were identified by SEB (lead author) and subsequently verified by GLJ/SJW (authors) and reduced to key ideas using a six stage process described below [9].

1. The data was transcribed from audio tape to a text file. The data was continually re-read and organised, allowing the researchers to become familiar with the data.

2. The data was categorised into themes by identification of recurring or underlying ideas that were pertinent to the interviewees.

3. The data was coded using the categories identified.

4. Challenges of the emerging themes were made by asking if the data (and themes) would answer any of the central questions of the study.

5. An alternative interpretation of the themes was sought, by investigating whether the data had a link to previous research.

6. The emergent themes from the data were then written-up.

## Results

Including the 4 HOPE who responded by mail and the remainder recruited via a snowball method, of the 40 teachers in total 43% (17/40) consented to be interviewed. Eleven teachers resided in the NW region and six teachers lived in the SW region of England. The interviewees consisted of five female HOPE and ten male HOPE. The least experienced HOPE had worked for 5 years in the role and the most experienced had worked for 25 years as a HOPE. The HOPE had worked in 30 schools collectively, with only two teachers having worked in just one school and two teachers having worked in four different schools. The overall response rate from HS was low with only two responding from 40 schools (5%). Of these, one HS was male from the NW of England and the other was female and resided in the SW. Together, both had over 20 years' teaching experience in secondary school education in England.

At the outset the intention was to gain a better understanding of the teachers' perspectives of provision and participation by pupils in physical activity and whether differences between regions (i.e. NW and SW) existed. However, as no differences between regions emerged a general picture of what the teachers thought about children's activity levels is provided. Where differences were identified between HS and HOPE these differences are discussed and quotes that do not include an interviewee number are representative of many of the study participants' comments.

After the six stage data analysis process the 17 interviews provided no new emerging themes and therefore it was concluded that data saturation had been reached. Of these 17 interviews, the longest lasted 1 hour and 10 minutes and the shortest 25 minutes (mean = 43 min in duration, SD 9.7 min).

There were 64 initial perceptions that arose from the HOPE and HS interviews. Seventeen themes emerged relating to encouraging PA or being a barrier to PA both inside and outside the curriculum (see Table 1). Of these results themes 1–8 related to barriers to PA and themes 9–17 related to encouraging PA. Twelve of the themes (Theme 1, 2, 3, 5, 8, 9, 10, 13, 14, 15, 16 & 17) collaborated findings of several previous research articles by different authors [2,5,10-20]. However, five themes had a novel or different viewpoint on the reasons behind adolescent's participation in PA (Theme 4, 6, 7, 11 & 12). The results of themes 1, 2, 3, 5, 8, 9, 10, 13, 14, 15, 16 & 17 are briefly described. The results of the newly identified themes: 4, 6, 7, 11 & 12 are described in more detail.

**Table 1:** Themes that emerged from interviews

**Theme number in text**	**Title of theme**	
	***Themes that echo similar research results in existing literature***	***Novel/new themes or themes offering a different viewpoint***
**1**	Time constraints	
**2**	Restricted curriculum	
**3**	HRE (health related exercise) confusion: exercise equals competition	
**4**		Ethos of performance/elitism within PE dept. or school as a whole
**5**	The lure of sedentary behaviour	
**6**		Lower fitness leads to lower ability
**7**		Undervaluing activities
**8**	Local authority's provision of leisure	
**9**	Ethos of PA for life within the school	
**10**	National curriculum: time and ownership	
**11**		Role of the school
**12**		PE department doing all it can
**13**	Provision of good facilities	
**14**	Agencies (e.g., local authority encourage the inactive)	
**15**	Appropriate funding of PA	
**16**	Self confidence in children	
**17**	Children's home and social environment	

### Barriers to PA in the curriculum

#### Theme 1: Time constraints

One strong topic to emerge, which was reported as a barrier to PA, was the time teachers were allocated at their respective schools for teaching PE. The vast majority of teachers felt the subject needed more curriculum time and felt that most other curriculum subjects were competing for this time. For example, very few schools had a policy whereby all pupils received a 2 hour core PE lesson per week. HOPE also felt that as pupils got older they had less opportunity to be active as evidenced by comments like *'definitely not enough [PE] in year ten and eleven.' *Conversely, the HS thought that the time allocated to PE and health-related exercise (this is a taught section of the national curriculum, delivered via practical and theoretical methods) in the curriculum was sufficient for pupils' activity.

#### Theme 2: Restricted curriculum

Some teachers' spoke of pupils having a limited input into the range of curriculum activities especially in key stage 3 (ages 11–14). Some schools still had a compulsory requirement of their key stage 4 (ages 15–16) pupils to study competitive team games, rather than alternative physical activities. This restricted opportunities for pupils to access 'lifetime' type activities particularly in the lower school years.

'... that's probably one of the major problems here [no 'lifetime' type activities]. It could be a lot better for the purpose of the kids that perhaps don't enjoy going out and playing football, that like to do maybe something on their own...' (Interviewee 7)

#### Theme 3: HRE (health related exercise) confusion: exercise equals competition

The results of the interviews indicate (from the teachers' perspective) that there was no standard method of delivery for HRE in schools. Consequently, there were inconsistencies regarding the way HRE was taught in the curriculum. For example, several teachers taught this as a block while others filtered HRE into PE lessons. The inconsistencies were highlighted by several HOPE comments like *'it does figure in PSHE *[Personal, Social and Health Education]*but its more by good luck than good judgement, its not like organised...' *and *'...health related fitness is purely PE's responsibility.' *The delivery of HRE raised mixed feelings among the teachers about which method was most effective in encouraging activity in the school curriculum. Some teachers practiced and favoured cross curricular HRE delivery whilst others favoured teaching HRE solely in the PE lesson. Sometimes teachers felt that HRE reinforced the idea to pupils that exercise equalled competition. This was especially true of the fitness testing elements often used within HRE. Some teachers seemed to have issues with this element, although they still relied on fitness testing as part of the curriculum.

#### Theme 4: Ethos of performance/elitism within PE dept. or school as a whole

HOPE often viewed there to be two competing interests within the PE department and this was a novel theme which emerged. Firstly, there was a desire to encourage elite performance and raise the profile of the school through sporting excellence. Yet, there was also a need to encourage mass participation in enjoyable, health-promoting physical activity. With regard to providing a broader programme of activities for less able pupils, one teacher stated:

'We would love to set up something like that, but it's the reality of if you still want to compete in the hockey, the netball, the rounders, the football, everything else, which is, we are one school in the Borough who will take part in every single activity that's going, you are struggling' (Interviewee 1)

In spite of this, some HOPE felt that although PE's overall message advocated activity for all pupils, some still felt strongly about keeping elitism:

*'... [I] make no excuses for wanting to produce excellence in particular sports.'*.....*'I see nothing wrong with it, other people think it's divisive and stops people playing, but that's one of those things.' (Interviewee 8)*

Others felt that certain schools, particularly those with a history of good performance in team events within their locality, would feel pressure to keep that success going. Therefore a conflict between providing activity for all and elite team performance would arise.

*'I can see for some teachers the time constraints of trying to sort out their extra curricular clubs or teams *(for the best reflection of the school through performance)*and things like that and using the PE lessons maybe to do that.' (Interviewee 5)*

Many interviewees looked at this as a dilemma concerning the aims of in school lessons against out of school clubs.

'As a teacher in lessons I think you want maximum [participation], but outside of lessons and the development of talent, I think you want to go elitist. So it's a bit of, that old dilemma.' (Interviewee 8)

#### Theme 5 & 6: The lure of sedentary behaviour and lower fitness leads to lower ability

A major influence affecting children personally were the distractions in life they had to draw them away from PA: *'I think these days children have a lot pulling at them...there are that many more opportunities for them....' *Most teachers expressed doubt that unless children had activity *'put on a plate for them' *they would not look for it *'...kids will not go out and proactively find it [PA]' *In addition, a potentially original finding emerged that several teachers were pessimistic about children's fitness and activity levels. Most HOPE thought that activity and fitness was lower in young people now compared to when they started out in teaching. Some felt this was possibly leading to lower ability of pupils *'...across the year group the sporting basic skills, throwing, catching are not as strong as they used to be*.' There was also a worry that amongst girls the decline was more dramatic.

Although the extra curricular opportunities afforded to pupils now were thought to be quite comprehensive, many HOPE thought that a lot of the same children would turn up to all the PA clubs:

'I always find that it's the same pupils that will do sport [clubs] and the same ones that wont attend any clubs.' (Interviewee 7)

This lead to several of the HOPE thinking the gap between active and sedentary children was getting wider; amongst the teachers who felt this, the comment below captured the disposition:

'One of the big things I have noticed now is that although you still get your top level performers being a similar standard to what they were years ago, there is now a big gap between those at the top, who do sport outside school and those sort of also ran's, shall we say.' (Interviewee 12)

### Barriers to PA outside the curriculum

Most schools now aim to provide activity opportunities outside curriculum hours in extra curricular clubs. The following two themes are factors that teachers felt had a discouraging effect on pupils PA outside the curriculum. Theme 7 is a novel/new theme specific to this study.

#### Theme 7: Undervaluing activities

Having support in extra curricular PE (e.g., lunches and after school) by other teachers, was by all accounts important in the smaller schools but tended not to happen. Therefore PA was being undervalued through this lack of volunteering by colleagues, as highlighted by this comment:*'I think extra curricular activity in schools has died a bit...' *explaining that in previous years teachers '*... were willing to give up their time at either lunch time or after school.'*

A number of teachers also made comments related to the school undervaluing activity or PE and a sense that senior management prioritised PE lower than other academic subjects. Interestingly, HS saw an aspect (if not the overall aim) of PE and HRE as 'recreation'. However, HS spoke of this as a benefit to health rather than their academic benefit, with one commenting that:

'...the opportunity to interact with other young people outside the classroom so they are actually in the fresh air you can see the difference... the difference between how the children are in the morning and how they are later on in the day when they have had PE and what have you at lunch time.' (HS 2)

#### Theme 8: Local authority's provision of leisure

Studies have examined the variables that determine young peoples PA and these variables were mentioned by HOPE several times [13]. One of these determinants was the availability and structure of local area institutions and organisations that influence young people's access to PA. HOPE consistently spoke of things like having transport to places that provide PA. Some teachers believed that community provision was good at schools but lacking outside of these venues. However, this was not unequivocal as some believed facilities at their school were poor. Despite this, most teachers agreed there was a lack of specific opportunities or facilities at existing venues in their area for young people to be physically active.

### Encouragement of PA in the curriculum

#### Theme 9: Ethos of PA for life within the school

HOPE spoke of numerous issues concerning the ethos of 'activity for life', whether this was for the whole school or specific to their department. Having said this, in the HS minds their school ethos was more tied into the specialism of the school e.g., languages, maths, arts or business: '*this school has got specific targets about raising attainment general targets, at key stage 3, key stage 4....' *Having a range of activities in the curriculum was again thought by many HOPE to encourage an 'activity for life' attitude. Some teachers reported a growing interest in these 'activities for life' as their pupils now preferred '*leisure type' *activities (i.e. aerobics, gym use) to the more traditional '*team games' *(i.e. hockey & football) and consequently more inactive pupils were attending extra curricular clubs. Many teachers felt an ethos of lifetime activity was the ultimate aim of the teaching of HRE and spoke passionately of those personal beliefs using words such as *'integrating children',...'inclusion',...'build self-esteem'...'engages them' *to describe this.

#### Theme 10: National curriculum: time and ownership

In contrast to the barriers to physical activity, the HOPE commented on several aspects which they felt encouraged activity in the curriculum. PE time again featured heavily. Those teachers whose year 10 and 11 pupils received 2 hours core PE (these were very few) were especially positive. The choice of activities in the curriculum was seen by some teachers as giving the pupils ownership over their learning and thus encouraged maximum participation:*'...we are trying to do lifelong learning so we introduce them to different activities in that sense, not necessarily competitive but just trying to broaden their horizons.' *However, a limited number of teachers did feel team games rather than alternative activities supported children's involvement in PA. With PE time being at such a premium, the HOPE felt they had to plan carefully to increase PA opportunity within school.

#### Theme 11 & 12: Role of the school and PE department doing all it can

In theme 11, the amount of exposure children have to activity within institutions was highlighted as a determinant of PA. Also a new emergent idea was that the schools role could directly influence the available curriculum time that was devoted to PA *'which school they go to can influence them' *was a common sentiment. This was inclusive of the schools general policies on physical activity to available PE lesson time and after school club provision. During the interviews, some HOPE made comments to suggest that the PE department was doing its utmost to maintain activity levels. The feeling amongst some of the teachers was that in lesson time children were as active as ever because the teacher would try to increase activity to make up for a general lack of activity outside of the PE environment.

### Encouragement of PA outside the curriculum

#### Theme 13, 14, 15, 16 & 17

Overall, the teachers reported five key themes that encourage PA outside of the curriculum in particular these were i) the provision of good facilities, ii) agencies, such as local authorities, encourage activity in children, iii) appropriate funding of PA, iv) self confidence in the school children and v) children's home and social environment. Facilities (which also came under authority's provision of leisure) were felt to be important by teachers. Teachers believed that having an abundance of facilities available in the school and community gave pupils more chances to be active. Some teachers felt external agencies provision of extra curricular clubs and alternative PA encouraged children who would not normally be active to take part. HOPE also felt that with a little improvement, institutions could better encourage PA through subsidy. Several HOPE thought public policy and availability of funding played an important part in contributing to an environment promoting young people's activity.

Further determinants of PA emerged under the theme of self-confidence. For example, teachers felt the pupil's upbringing would affect their confidence *'... I really do think its how they are brought up. If they are used to being active they develop the skills they need, so then they are more confident when they use them....' *The teachers suggested that the social and home environment the child was immersed in would have a direct bearing on their PA level e.g. their friends, peers and family environment. Certainly, in this set of interviews all HOPE and HS felt parents were a major influence in pupil's activity and mentioned this frequently, typical comments were: *'its got to be parents [biggest influence on pupil's PA].' 'Parents I think that has a huge, huge impact. The lifestyle of the parent, their support to take children out.'*

## Discussion

From the 17 themes identified, five offered novel reasons behind adolescents participation in PA (Theme 4, 6, 7, 11 & 12) while 12 (Theme 1, 2, 3, 5, 8, 9, 10, 13, 14, 15, 16 & 17) confirmed findings from other studies [2,5,10-20].

A novel theme or issue that arose in several (but not all) of the HOPE's interviews was the 'ethos of elitism'. In this study some HOPE thought elitism was essential in order to promote and maintain the school's prestige within the local area. Consequently, they believed that only gifted and talented pupils were mainly encouraged to participate in PA, often at the expense of mass participation and those less able pupils. Several papers have reported that team games are favoured in the curriculum over alternative or 'lifetime' activities [5,10,11] and it could be argued the HOPE's thoughts were an extension of this. Several HOPE spoke of wanting to deliver a wider range of PA, certainly in extra curricular time, but had no evening free that didn't already host a team sport. This raised an issue that while the government 'throws its weight behind' initiatives to increase young people's activity (e.g., PE and School Sport Club Links, active schools, healthy schools [3]) the child who wants to be active but does not like team games may be alienated. A lot of PA provision in this study (certainly that of extra curricular activity) was through competitive team games, which could be providing for a minority of 'sporty' pupils only. It was interesting that some HOPE were prepared to encourage elitism but still wanted all children in general to be active and fit (often through aspects of HRE). These HOPE felt out of school clubs should be concerned with performance or provision for school teams whereas lessons should promote activity for all.

Although most HOPE and HS in this study thought that over the course of their teaching career children's activity levels had fallen, research into the area has failed to conclusively confirm this. For example, Sport England (2000) [21] and Balding (2004) [22] both reported a slight increase in children's activity. However, several other reviews have examined the issue of children and adolescents activity levels and reported that whilst adolescents in general were more active than adults these levels were not sufficient enough to benefit health.

Besides activity, the HOPE had concerns with pupils' fitness levels and were convinced that they had fallen due to less PA being undertaken, something Kulinna and Silverman concur with [23]. Similar was true of the teachers in Cale's study [24] who clearly thought promotion of activity was important because children are currently not as active or fit as they were in the past. Comparable findings were reported in Green and Thurston [11]. Teachers in their study felt young people now engaged in a lack of activity and had a lack of fitness. A majority of HOPE expressed concern about pupil's activity decreasing further as they became older. This finding has been supported by other literature in this area [13,18] and possibly justifies the concerns raised by some HOPE that the schools were sending the wrong message by reducing PE time in key stage 4. We found this to be of more concern in girls: a finding which has also been reported in several other studies and reviews in this area [25-33].

Interestingly, although many of the views of HOPE in this study regarding pupils fitness have been reported previously, the idea that this 'lack of fitness' was leading to lower ability levels in pupils is possibly a new one. Several HOPE thought there was now a discernable difference in basic skill levels between those pupils active outside school and those not. This could be a worrying reflection of the Primary school provision of activity and PE and younger children's play habits outside school. An interesting finding of the HOPE and one which has arisen in previous studies of activity in this population [12,13,34], was the feeling that, at present, adolescents are faced with many more choices of what to do with their leisure time and given the option would choose to be sedentary.

Under theme 9 (ethos of PA for life ...) the HOPE spoke of several aspects within the school environment promoting an ethos of activity, ideas backed in other research [35-39]. However, two themes, number 7 (undervaluing activities) and 11 (role of the school) which were closely linked to the previous findings offered a slightly different view these HOPE took of the provision of activity for life. As a barrier to PA several HOPE felt the school undervalued PE and sensed that PE was a low priority to senior management. A consequence of this was that maybe pupils' activity in that particular school would be seen as less important. Something that Cale [24] found was that teachers may have a narrow view of promoting PA throughout the schools ethos not just in the PE department or PE lesson.

Interestingly, although the HS interviewed recognised the value of PA to health, it was thought of as a recreational pursuit not an academic one. However, teachers' spoke of the important role school played in influencing pupils' activity. Many of the teachers in Green and Thurston [11] also felt a key role of the PE teacher was the promotion of an active lifestyle, thus backing the HOPE's thinking that a school not showing an interest in PA would be detrimental. Therefore, the HOPE felt this gave the individual school itself more responsibility in placing activity as a high priority. One policy the government have recently introduced to increase activity is that all schools should aim for two hours provision of 'quality PE and sport' for all its pupils [3]. Most teachers had an issue with this, thinking the idea was somewhat misleading as the provision could be made up of taught lessons and extra curricular activity, which not all pupils may have access to. Therefore, a strong argument would be to make the two hours provision purely within taught curriculum PE. HOPE in this study were certainly concerned that older children (years 10 and 11) did not get access to this two hours in the curriculum and because not every child can attend extra curricular activity few would make up this time shortage after school.

Having said this, several HOPE in this study believed that the PE department was doing all it could to address children's activity levels. One explanation for this could be due to the wider range of activities that have gradually been filtering into the 'traditional' PE curriculum [15,24,40]. Also some believed the levels of activity in the PE lesson were as high as ever. However, the HOPE as discussed previously believed children's fitness had fallen. This idea could be similar to Khan et al [17] systematic review findings from intervention studies that altered the delivery of the PE programme. Although none of the Khan et al [17] studies were UK based, the ones that modified the PE programme showed increases in activity at school [41,42].

Nevertheless, few studies in Khan et al [17] showed increases in PA outside of school and one study [43] by Donnelly and colleagues found that while activity in school had increased, out of school activity was actually lower in the intervention group. Even though most of the HOPE's extra curricular programmes seemed to offer some deviation away from team games, it was only mainly the pupils who were gifted and talented at sport that were choosing to participate, a finding which has been hinted at in other studies [11,29,32,33].

The study has several limitations, firstly SEB (lead author) is a former PE teacher familiar with PE issues and we must recognise that this familiarity may have had consequences for the data collection (interviews) and data analysis. To ameliorate this affect, SEB endeavoured to be impartial during the interviews and data analysis. During the data analysis the emergent themes were checked by the two other authors (GLJ and SJW). Secondly, HOPE in the study were not evenly distributed, there were five from the SW and ten from the NW so the themes may not be representative of the two areas. Although data saturation was also reached and no additional new themes emerged, the study size and number of interviews (n = 17) may be a limitation; particularly for the HS sample. The HS had a low response rate (only 5% from both areas responded). Therefore, interviewing more school heads may have led to a greater understanding of children's PA levels from their perspective and it is an area we recommend for future research. In addition, more research (possibly quantitative to gauge perspectives across several regions) into PE's delivery, its influence on children's PA and a wider scope of regions in England would also be worthwhile and is a recommended area of future research.

## Conclusion

In the light of the recent policies and initiatives giving physical activity increased importance in the school's curriculum, the findings from this study revealed inconsistencies. Whilst improvement is needed in the delivery of PE and activity promotion and the government has strived to increase provision of this activity, HOPE still felt that the allocated time for PE was too little. Also, although more alternative and lifetime activities were filtering into the curriculum in schools (this was still very slow), it remained largely extra curricular. The delivery of health-related exercise within the curriculum, as research in the past has also highlighted, is not consistent and confusion over its delivery exists. The thoughts of the HOPE in their assessment of pupil's fitness levels are perhaps reflected in pupil's willingness to be sedentary rather than active. It is clear the HOPE place an enormous amount of responsibility of children's activity at the feet of the parents. Hence, it would be interesting to find out the perspective of the inactive child's parents and this may provide another possible avenue of research.

An ethos of 'activity via policy and practice' must be built into a school, its community or even the whole local authority in order to influence young people's desire to be active. This would help create not just a physical but a social environment to actively promote activity. However, from the perspective of the teacher's interviewed in this study it was still not happening. Despite a feeling of promoting 'activity for life' to pupils it was apparent that some HOPE were clear that aspects of elitism were important for them sometimes to the cost of mass participation by less able pupils. This is an area for possible investigation especially in light of the pending Olympics in 2012, which may press teachers even more to strive for elitism of the few.

## Competing interests

The authors declare that they have no competing interests.

## Authors' contributions

SEB assisted with the design of the methodology, carried out data collection, analysis and prepared the manuscript for submission. GLJ and SJW assisted with the design of the methodology, assisted analysis and writing of the manuscript. All authors read and approved the final manuscript.

## Pre-publication history

The pre-publication history for this paper can be accessed here:


